# Supplementary material for: The impact of environmental enrichment on energy metabolism in ovariectomized mice
Source: PLoS One. 2025 Apr 2;20(4):e0320180. doi: 10.1371/journal.pone.0320180 (PMC11964458; doi:10.1371/journal.pone.0320180)
Supplement: Attachment — Submitted filename: PONE-D-25-01987.pdf [file pone.0320180.s002.pdf]

PLOS ONE

The impact of environmental enrichment on energy metabolism in ovariectomized mice

--Manuscript Draft--

**Manuscript Number:**	PONE-D-25-01987
**Article Type:**	Research Article
**Full Title:**	The impact of environmental enrichment on energy metabolism in ovariectomized mice
**Short Title:**	Environmental Enrichment Improves Metabolic Parameters in Menopause Model
**Corresponding Author:**	Eijiro Jimi<br>Kyushu University OBT Research Center<br>Fukuoka, JAPAN
**Keywords:**	Environmental enrichment, menopause, ovariectomized model, energy metabolism, circadian rhythm
**Abstract:**	After menopause, a decline in ovarian function leads to various physical and psychological changes, potentially resulting in a range of pathological conditions, including abnormalities in energy metabolism. In recent years, environmental enrichment, which is characterized by positive and comfortable eustress, has been shown to improve various physiological and pathological conditions. This study investigated the effects of environmental factors on energy metabolism in a menopause model using an ovariectomized (OVX) mouse model. Wild-type female mice (8-week-old) were subjected to OVX or a sham operation and maintained under standard (SC), enriched (EE), or isolated (IS) conditions for 4 weeks. OVX led to weight gain and disruption of circadian rhythms, along with changes in various metabolic parameters influenced by differences in housing environments; i.e., EE improved metabolic parameters, but IS deteriorated them. Physical activity and social interaction were factors that determined these differences. Menopause is usually a significant transitional period in a woman's life, and changes in the social environment during this period can contribute to a diverse range of physical and psychological symptoms. Consequently, when implementing interventions to alleviate menopause-related pathological conditions, not only physical symptoms but also the social context should be carefully considered.
**Order of Authors:**	Chaoran Ju, DDS.
	Ayano Ogura, DDS., Ph.D.
	Yoshikazu Hayashi, DDS, Ph.D.
	Yuko Kawabata, Ph.D.
	Fulvio D'Acquisto, Ph.D.
	Tomoyo Kawakubo-Yasukochi, DDS, Ph.D.
	Eijiro Jimi, DDS,Ph.D.
**Additional Information:**	
**Question**	**Response**
**Financial Disclosure**<br><br>Enter a financial disclosure statement that describes the sources of funding for the work included in this submission. Review the submission guidelines for detailed requirements. View published research articles from *PLOS ONE* for specific examples.	Yes

Powered by Editorial Manager® and ProduXion Manager® from Aries Systems Corporation

This statement is required for submission and **will appear in the published article** if the submission is accepted. Please make sure it is accurate.<br><br>**Funded studies**<br>Enter a statement with the following details:<br>• Initials of the authors who received each award<br>• Grant numbers awarded to each author<br>• The full name of each funder<br>• URL of each funder website<br>• Did the sponsors or funders play any role in the study design, data collection and analysis, decision to publish, or preparation of the manuscript?<br><br>Did you receive funding for this work?	
Please add funding details.<br>as follow-up to "**Financial Disclosure**<br><br>Enter a financial disclosure statement that describes the sources of funding for the work included in this submission. Review the submission guidelines for detailed requirements. View published research articles from *PLOS ONE* for specific examples.<br><br>This statement is required for submission and **will appear in the published article** if the submission is accepted. Please make sure it is accurate.<br><br>**Funded studies**<br>Enter a statement with the following details:<br>• Initials of the authors who received each award<br>• Grant numbers awarded to each author<br>• The full name of each funder<br>• URL of each funder website<br>• Did the sponsors or funders play any role in the study design, data collection and analysis, decision to publish, or preparation of the manuscript?	the Japan Society for the Promotion of Science (JSPS) Grants-in-Aid for Scientific Research (KAKENHI) (Grant No. 20KK0213 to EJ).

Did you receive funding for this work?"	
Please select the country of your main research funder (please select carefully as in some cases this is used in fee calculation).<br>as follow-up to "**Financial Disclosure**<br><br>Enter a financial disclosure statement that describes the sources of funding for the work included in this submission. Review the submission guidelines for detailed requirements. View published research articles from *PLOS ONE* for specific examples.<br><br>This statement is required for submission and **will appear in the published article** if the submission is accepted. Please make sure it is accurate.<br><br>**Funded studies**<br>Enter a statement with the following details:<br>• Initials of the authors who received each award<br>• Grant numbers awarded to each author<br>• The full name of each funder<br>• URL of each funder website<br>• Did the sponsors or funders play any role in the study design, data collection and analysis, decision to publish, or preparation of the manuscript?<br><br>Did you receive funding for this work?"	JAPAN - JP
**Competing Interests**<br><br>Use the instructions below to enter a competing interest statement for this submission. On behalf of all authors, disclose any competing interests that could be perceived to bias this work—acknowledging all financial support and any other relevant financial or non-financial competing interests.<br><br>This statement is required for submission and **will appear in the published article** if	The authors have declared that no competing interests exist.

the submission is accepted. Please make sure it is accurate and that any funding sources listed in your Funding Information later in the submission form are also declared in your Financial Disclosure statement.<br><br>View published research articles from *PLOS ONE* for specific examples.<br><br>**NO authors have competing interests**<br><br>Enter: *The authors have declared that no competing interests exist.*<br><br>**Authors with competing interests**<br><br>Enter competing interest details beginning with this statement:<br><br>*I have read the journal's policy and the authors of this manuscript have the following competing interests: [insert competing interests here]*<br><br>* typeset	
**Ethics Statement**<br><br>Enter an ethics statement for this submission. This statement is required if the study involved:<br><br>• Human participants<br>• Human specimens or tissue<br>• Vertebrate animals or cephalopods<br>• Vertebrate embryos or tissues<br>• Field research<br><br>Write "N/A" if the submission does not require an ethics statement.<br><br>General guidance is provided below. Consult the submission guidelines for detailed instructions. **Make sure that all information entered here is included in the Methods section of the manuscript.**	All animal experiments were approved by the Animal Care Committee of Kyushu University (approval numbers A21-358 and A23-168).

**Format for specific study types**<br><br>**Human Subject Research (involving human participants and/or tissue)**<br>• Give the name of the institutional review board or ethics committee that approved the study<br>• Include the approval number and/or a statement indicating approval of this research<br>• Indicate the form of consent obtained (written/oral) or the reason that consent was not obtained (e.g. the data were analyzed anonymously)<br><br>**Animal Research (involving vertebrate animals, embryos or tissues)**<br>• Provide the name of the Institutional Animal Care and Use Committee (IACUC) or other relevant ethics board that reviewed the study protocol, and indicate whether they approved this research or granted a formal waiver of ethical approval<br>• Include an approval number if one was obtained<br>• If the study involved *non-human primates*, add *additional details* about animal welfare and steps taken to ameliorate suffering<br>• If anesthesia, euthanasia, or any kind of animal sacrifice is part of the study, include briefly which substances and/or methods were applied<br><br>**Field Research**<br><br>Include the following details if this study involves the collection of plant, animal, or other materials from a natural setting:<br>• Field permit number<br>• Name of the institution or relevant body that granted permission	
**Data Availability**<br><br>Authors are required to make all data underlying the findings described fully available, without restriction, and from the time of publication. PLOS allows rare exceptions to address legal and ethical concerns. See the PLOS Data Policy and FAQ for detailed information.	Yes - all data are fully available without restriction

A Data Availability Statement describing where the data can be found is required at submission. Your answers to this question constitute the Data Availability Statement and **will be published in the article**, if accepted.<br><br>**Important:** Stating 'data available on request from the author' is not sufficient. If your data are only available upon request, select 'No' for the first question and explain your exceptional situation in the text box.<br><br>Do the authors confirm that all data underlying the findings described in their manuscript are fully available without restriction?	
**Describe where the data may be found in full sentences. If you are copying our sample text, replace any instances of XXX with the appropriate details.**<br><br>• If the data are **held or will be held in a public repository**, include URLs, accession numbers or DOIs. If this information will only be available after acceptance, indicate this by ticking the box below. For example: *All XXX files are available from the XXX database (accession number(s) XXX, XXX.*).<br>• If the data are all contained **within the manuscript and/or Supporting Information files**, enter the following: *All relevant data are within the manuscript and its Supporting Information files.*<br>• If neither of these applies but you are able to provide **details of access elsewhere**, with or without limitations, please do so. For example:<br><br>*Data cannot be shared publicly because of [XXX]. Data are available from the XXX Institutional Data Access / Ethics Committee (contact via XXX) for researchers who meet the criteria for access to confidential data.*<br><br>*The data underlying the results presented in the study are available from (include the name of the third party*	All relevant data are within the manuscript.

and contact information or URL).<br>• This text is appropriate if the data are owned by a third party and authors do not have permission to share the data.<br><br>* typeset	
Additional data availability information:	Tick here if the URLs/accession numbers/DOIs will be available only after acceptance of the manuscript for publication so that we can ensure their inclusion before publication.

Powered by Editorial Manager® and ProduXion Manager® from Aries Systems Corporation

Manuscript Click here to access/download;Manuscript;Ju C et al. Text.docx

The impact of environmental enrichment on energy metabolism in ovariectomized mice

Chaoran Ju[1], Ayano Ogura[1,2], Yoshikazu Hayashi[3,4,5], Yuko Kawabata[6], Fulvio D'Acquisto[7,8], Tomoyo Kawakubo-Yasukochi[3], Eijiro Jimi[1,3]

[1]Laboratory of Molecular and Cellular Biochemistry, Division of Oral Biological Sciences, Faculty of Dental Science, Kyushu University, 3-1-1 Maidashi, Higashi-ku, Fukuoka 812-8582, Japan

[2]Department of Cell Biology, Aging Science, and Pharmacology, Division of Oral Biological Sciences, Faculty of Dental Science, Kyushu University, 3-1-1 Maidashi, Higashi-ku, Fukuoka, Japan

[3]Oral Health/Brain Health/Total Health Research Center, Faculty of Dental Science, Kyushu University, 3-1-1 Maidashi, Higashi-ku, Fukuoka 812-8582, Japan.

[4]Division of Functional Structure, Department of Morphological Biology, Fukuoka Dental College, 2-15-1 Tamura, Sawara-ku, Fukuoka 814-0193, Japan.

[5]Oral Medicine Research Center, Fukuoka Dental College, Fukuoka 814-0193, Japan

[6]Section of Oral Neuroscience, Division of Oral Biological Sciences, Faculty of Dental Science, Kyushu University, 3-1-1 Maidashi, Higashi-ku, Fukuoka, 812-8582, Japan.

[7]School of Life and Health Science, University of Roehampton, London, SW15 5P, United Kingdom.

[8]William Harvey Research Institute, School of Medicine and Dentistry, Queen Mary University of London, London, EC1M 6BQ, United Kingdom.

Corresponding authors: Tomoyo Kawakubo-Yasukochi, DDS, PhD, and Eijiro Jimi, DDS, PhD, Oral Health/Brain Health/Total Health Research Center, Faculty of Dental Science, Kyushu University, 3-1-1 Maidashi, Higashi-ku, Fukuoka 812-8582, Japan, Tel: +81-92-642-6332, E-mail: tomoyo@dent.kyushu-u.ac.jp, ejimi@dent.kyushu-u.ac.jp

Abstract

After menopause, a decline in ovarian function leads to various physical and psychological changes, potentially resulting in a range of pathological conditions, including abnormalities in energy metabolism. In recent years, environmental enrichment, which is characterized by positive and comfortable eustress, has been shown to improve various physiological and pathological conditions. This study investigated the effects of environmental factors on energy metabolism in a menopause model using an ovariectomized (OVX) mouse model. Wild-type female mice (8-week-old) were subjected to OVX or a sham operation and maintained under standard (SC), enriched (EE), or isolated (IS) conditions for 4 weeks. OVX led to weight gain and disruption of circadian rhythms, along with changes in various metabolic parameters influenced by differences in housing environments; i.e., EE improved metabolic parameters, but IS deteriorated them. Physical activity and social interaction were factors that determined these differences. Menopause is usually a significant transitional period in a woman's life, and changes in the social environment during this period can contribute to a diverse range of physical and psychological symptoms. Consequently, when implementing interventions to alleviate menopause-related pathological conditions, not only physical symptoms but also the social context should be carefully considered.

Keywords: Environmental enrichment, menopause, ovariectomized model, energy metabolism, circadian rhythm

***Abbreviations*:** OVX: ovariectomy; SC: standard condition; EE: enriched environment; IS: isolated condition; gWAT: gonadal white adipose tissue; ELISA: enzyme-linked immunosorbent assay; H&E: Mayer's hematoxylin and eosin Y; GTT: glucose tolerance test; PTT: pyruvate tolerance test; HOMA-IR: Homeostatic Model Assessment for Insulin Resistance; AUC: area under the curve.

Introduction

It has been suggested that the living environment may affect not only the physiological status [1] but also the progression of diseases [2], although environmental enrichment in humans remains controversial and is difficult to analyze in detail because there is no standardized environment. In laboratory animals, a housing environment that includes social interaction and physical exercise in space use is called environmental enrichment, and it has been reported to be effective in eliciting animals' innate behavior and improving behavior [3], neurological functions [3], energy metabolism [4], and cancer progression [4, 5]. Therefore, the living environment has been recognized as a research subject [3].

Along with the changing social structure, households are becoming smaller and aging, and the number of elderly people living alone is increasing in Western countries and Japan [6]. Living alone increases the risk of social isolation and shortens healthy life expectancy [7]. Therefore, there is an urgent need to present scientific evidence that living environment is related to the onset or progression of the disease to propose strategies for effective interventions and disease prevention. In particular, postmenopausal women are known to have an increased risk of developing various lifestyle-associated diseases originally regulated by estradiol, such as osteoporosis, obesity, and heart disease [8]. However, no detailed studies have examined the influence of environmental factors on various diseases in postmenopausal women. Therefore, in this study, we investigated whether environmental factors, such as environmental enrichment or isolation, affect energy metabolism abnormalities after menopause.

Material and methods

Mice

All animal experiments were approved by the Animal Care Committee of Kyushu University (approval numbers A21-358 and A23-168).

Experimental animals and environmental conditions

C57BL/6J female mice, obtained from CLEA Japan (Tokyo, Japan), were raised in specific-pathogen-free facility under controlled environmental conditions (a 12-hours light/dark cycle, temperature of 20–26 °C, and humidity of 40–70%). The mice were randomly assigned at 8-week-old to either the enriched environment (EE), the standard condition (SC), or the isolated condition (IS). The EE mice (5 mice/cage) were housed in a cage (267[W] × 426[D] × 150[H] mm) consisted of a running wheel and shelter (Mouse lgloo and Fast Tracs, Bio-Serv, Flemington, NJ, USA) and a tunnel (Shepherd Tube, EP Trading, Tokyo, Japan), in which a bedding mixture of 2:1 soft bedding (Alpha-dri-Certified, EP Trading) and woody tips (Tokojiki, Douourika, Sapporo, Japan) was used. The SC (5 mice/cage) and IS mice (1 mouse/cage) were maintained in standard cages (168[W] × 299[D] × 133[H] mm) with no additional objects, in which only woody tips (Tokojiki, Douourika) were used. All bedding in the cages was replaced weekly. The mice were either sham-operated (sham) or ovariectomized (OVX) at 9 weeks of age under anesthesia using intraperitoneal injections of 0.75 mg/kg medetomidine, midazolam (4 mg/kg), and saline (5 mg/kg) [9].

Histological analysis

White adipose tissue was fixed with 4% paraformaldehyde and embedded in paraffin. For histological examination, Mayer's hematoxylin and eosin Y staining was applied to each 5 µm-thick section and observed using a microscope (BZ-X800, KEYENCE, Osaka, Japan). The adipocyte area was measured using the BZ-X800 Analyzer software (KEYENCE).

Glucose (GTT) and pyruvate (PTT) tolerance tests

At 12 weeks of age, the mice were subjected to an intraperitoneal injection of glucose (2 mg/kg body weight) or pyruvate solution (Nacalai Tesque, Kyoto, Japan, 1.5 g/kg body weight) after 20 hours of fasting. Glucose levels were assessed using a handheld glucometer (Freestyle Freedom Lite, Abbott Park, IL, USA) at 0, 15, 30, 60, and 90 minutes after injection.

Homeostasis model assessment of insulin resistance (HOMA-IR)

The HOMA-IR index was calculated using Equation (1) [10]:

$$\text{HOMA-IR} = \text{fasting serum glucose (mg/dl)} \times \text{fasting serum insulin (ng/ml)} \times 26/405 \quad (1)$$

Serum glucose and insulin levels after fasting for 20 hours were measured using a D-glucose assay kit (GOPOD Format; Megazyme, Wicklow, Ireland) and an LBIS™ Mouse Insulin ELISA Kit (U-type) (Fujifilm Wako, Osaka, Japan), respectively.

Serum corticosterone measurement

Serum corticosterone levels were measured using the DetectX Corticosterone ELISA Kit (Arbor Assays, Ann Arbor, MI, USA) according to the manufacturer's instructions.

Serum biochemical analysis

All serum biological analysis was performed by Oriental Yeast (Tokyo, Japan).

Measurement of mouse momentum and body temperature

The amount of activity and subcutaneous temperature in the mice were monitored using an implantable device (nanotag, Kissei Comtec, Matsumoto, Japan) and analyzed using the nanotag Viewer program (Kissei Comtec) [11].

Measurement of daily food consumption

Daily feed intake was measured using a powder feeder and multifeeder (MF3S for an IS cage and MF4S for SC and EE cages) (SHINFACTORY, Fukuoka, Japan).

Statistical analysis

Statistical analyses were performed using GraphPad Prism version 10.2.2 (GraphPad Software, San Diego, CA, USA). The specific symbols for *P*-values are defined as

follows: *$P < 0.05$, **$P < 0.01$, ***$P < 0.001$, and ****$P < 0.0001$.

Results

Effects of body weight change and housing environment in sham and OVX mice. To evaluate the effects of different environments on sham and OVX menopausal model mice, mice were randomly assigned to SC, EE, and IS environments to allow them to acclimate to the environment one week before the operation (sham or OVX) (Fig. 1A,B). At the time of the sham/OVX operation (week 1), no difference in body weight was observed among the groups (Fig. 1C). Sham and OVX mice raised for an additional 3 weeks (week 4) showed no marked differences in body weight between the SC, EE, and IS environments (Fig. 1D). OVX-induced weight gain was observed in each housing environment (Fig. 1D), which was consistent with a previous report that OVX mice had higher body weights than sham-operated mice [12]. The difference in body weight gain between weeks 1 and 4 also indicated that OVX led to weight gain regardless of the environment (Fig. 1E).

Effects of housing environment on feed intake, energy expenditure, and body temperature in sham-operated and OVX mice. Body weight gain is closely related to an energy imbalance between energy intake and expenditure [13]. Therefore, we investigated whether there were differences in feed intake, physical activity, and body temperature between these mice in the different environments. Daily feed intake was monitored during week 3. Regardless of whether they were in the sham or OVX group, the feed intake of SC mice was substantially higher than that of EE mice (Fig. 2A). In addition, OVX led to a higher increase in feed intake in IS than in EE mice (Fig. 2A). In the sham group, the physical activity level of EE mice in week 3 was substantially higher than that in the SC and IS groups; however, that in the EE-sham mice was markedly lower after OVX (Fig. 2B,C), which is consistent with the fact that the decrease in estradiol reduces physical activity [14]. Estradiol regulates circadian rhythm [14]. The circadian rhythm of physical activity observed in sham mice, which indicates

decreased activity during the light period and increased activity during the dark period, was not clearly observed in the OVX group (Fig. 2B,C).

The circadian rhythm of body temperature, which is a vital metabolic sign, was also disrupted in the OVX group (Fig. 2D). In addition, a marked decrease in body temperature was observed in the IS-sham group compared to that in the EE-sham group, especially during the light period (Fig. 2E–G). No changes in body temperature were observed in the OVX groups in the housing environments (Fig. 2D–G).

Effects of housing environment on adipose tissue phenotype in sham-operated and OVX mice. To investigate the cause of weight gain in OVX mice (Fig. 1E), gonadal white adipose tissue (gWAT) was analyzed 4 weeks after environmental habituation (weeks 5, Fig. 1A). A substantial increase in gWAT mass in OVX mice compared to that in sham mice in each corresponding environment was observed (Fig. 3A), suggesting that OVX-related body weight change was closely related to gWAT weight change. Furthermore, gWAT weight was substantially lower in EE mice than in the sham and OVX groups (Fig. 3A), whereas no change in body weight was found in the latter groups (Fig. 1C–E). This tendency was negatively correlated with physical activity (Fig. 2B,C), suggesting that exercise might alter the quantity and quality of gWAT. The histological analysis indicated that the adipocytes of EE mice were substantially smaller than those of SC mice in the sham and OVX groups (Fig. 3B,C), and there was a positive correlation between gWAT weight (Fig. 3A) and adipocyte size (Fig. 3B,C). These results suggest that gWAT expansion was mainly caused by adipocyte hypertrophy.

Effects of housing environment on glucose metabolic parameters in sham and OVX mice. Since white adipose tissues play an essential role in glucose homeostasis [15, 16], glucose metabolism disorders are commonly found during menopause [17]. Therefore, the effects of the environment on glucose metabolic parameters in sham and OVX mice were investigated.

In glucose tolerance tests (GTT), IS-sham mice showed decreased glucose tolerance compared to mice in EE and SC, although there was no statistical difference between sham and OVX mice in the IS environment (Fig. 4A,B). The most characteristic trend was an increase in blood glucose levels 15 minutes after glucose injection and fasting glucose levels under IS conditions in both sham and OVX mice (Fig. 4C,D), although there was no difference in leisure blood glucose levels among the groups (Fig. 4E). Severe postprandial glucose spike and high fasting glucose levels adversely affect long-term glycemic control and increase the risk of cardiovascular events, and which can lead to more damage than a steady state of high glucose [18-20].

Menopausal mouse models exhibit insulin resistance and hyperinsulinemia [21]. In the current study, the leisure serum insulin concentration in OVX mice was substantially higher than that in sham mice in SC and IS, and this tendency was improved in EE (Fig. 1F). In contrast, no marked difference was observed in fasting serum insulin levels between sham and OVX mice (Fig. 4G), as previously reported [22], but it was decreased in EE compared to IS in the OVX groups (Fig. 4G).

HOMA-IR, an index of insulin resistance calculated from fasting serum glucose and fasting insulin concentrations, was substantially elevated in the IS compared to EE in the OVX mice (Fig. 4H).

A strong relationship exists between glucose regulation and glucocorticoids [23], which are the main effectors of stress response [23, 24]. A study reported that OVX increased serum glucocorticoid content compared to sham-operated mice [25], but there is a report of no change in these contents [26]. In the current study, we observed an OVX-induced increase in corticosterone levels in the EE and IS groups, although there was no change in serum corticosterone content due to environmental differences (Fig. 4I). Serum corticosterone contents in mice other than EE-sham mice increased over the reference range, suggesting that these mice might be in a stressful environment [27].

Gluconeogenesis is partially regulated by glucocorticoids [28-30]; therefore, gluconeogenesis was studied in in the mice. Glycemic excursion in response to pyruvate during pyruvate tolerance tests (PTT) was suppressed in the EE compared to the IS

environment in the sham and OVX groups (Fig. 4L,M).

Serological test results showed no statistical differences between the groups in total protein, albumin, LDL-cholesterol (LDL-C), HDL-cholesterol (HDL-C), total cholesterol (T-CHO), Sodium (Na), Chloride (Cl), Calcium (Ca), and Inorganic phosphorus (IP), whereas triglyceride (TG) was substantially increased in SC compared EE and IS groups in OVX mice (Fig. 4N–W). In addition, the serum potassium levels were markedly lower in IS-OVX than in EE-OVX mice (Fig. 4U). Given that insulin is a regulator of potassium intracellular uptake [31], high serum insulin concentrations (Fig. 4G,F) can be correlated with decreased serum potassium levels in IS-OVX mice (Fig. 4U).

Discussion

In the current study, we proved that OVX induced weight gain and circadian rhythm disruption, and that metabolic parameters changed depending on the housing environment; EE was better and IS was worse. Therefore, glucose intolerance and insulin resistance were the most affected in IS-OVX mice.

Exercise is an important environmental factor. We suggest that exercise in EE induces increased muscle mass and fat burning, and improves metabolic parameters. Although the space allocation per mouse was greater in the IS than in the SC environment, there was no difference in physical activity levels between the SC and IS. The reason for the deterioration in energy metabolism in IS-OVX mice over SC-OVX mice was likely due to decreased social enrichment. Although housing densities are also known to be important for animal welfare [32], social isolation had a major impact on metabolic abnormalities in IS-OVX mice in this study.

No definitive correlations were found between feed intake, exercise, and body weight. It is generally known that overeating and a lack of exercise are not direct causes of body weight gain [33], and that feed intake, physical activity, and body weight changes are influenced by multiple factors, including stress [33], such that further studies on endocrine factors are required. The changes in body weight and gWAT were not always

correlated in all groups. Changes in fat mass also affect changes in lean muscle mass. Exercise promotes an increase in muscle mass [34] and fat burning [35]; changes in both muscle and fat mass lead to changes in body weight. Therefore, further analyses of body composition may be required to determine whether changes in body weight are dependent on fat or lean mass in all mice.

Furthermore, changes in body temperature owing to stress have long been reported [36]. In the current study, body temperature was substantially lower in IS-sham than in EE-sham mice (Fig. 2E–G), but no differences in corticosterone levels were observed between the groups (Fig. 4I). The temperature of the experimental room was set to 20–26 °C, in accordance with international guidelines [37]. However, this temperature environment can be cold stress for rodents, in which the body temperature can easily drop if they are constantly active [37]. Therefore, body temperature results can be attributed to the differences between groups and individual housing.

Conclusion

This study demonstrated that in a housing environment in which exercise and social contact are eliminated, energy metabolism abnormalities can occur within a short period of time. OVX disrupts circadian rhythms, resulting in a decrease in physical activity even in an enriching environment; social isolation worsens metabolic abnormalities such as insulin resistance. Based on this evidence, we suggest that an enriched living environment with societal contact and activity are important for promoting health, especially after menopause.

Acknowledgements

This work was supported by grants from the Japan Society for the Promotion of Science (JSPS) Grants-in-Aid for Scientific Research (KAKENHI) (Grant No. 20KK0213 to EJ).

Author Contributions

Chaoran Ju: Methodology, Investigation, Writing – original draft. **Ayano Ogura:** Investigation. **Yoshikazu Hayashi:** Investigation, Statistical processing, Writing. **Yuko Kawabata:** Investigation, Statistical processing, Writing. **Fulvio D'Acquisto:** Conceptualization, Methodology, Writing – original draft, review & editing, **Tomoyo Kawakubo-Yasukochi**: Investigation, Writing – original draft, review & editing. **Eijiro Jimi:** Funding acquisition, Conceptualization, Methodology, Writing – original draft, Writing – review & editing.

Declaration of competing interests

The authors have declared no conflict of interest.

Data availability

All data are fully available without restriction.

References

1. Simone-Freilicher E, Rupley AE. Juvenile psittacine environmental enrichment, Vet Clin North Am Exot Anim Pract. 18 2015; 18:213-231. doi: 10.1016/j.cvex.2015.01.003 PMID: 25902270

2. McDonald MW, Hayward KS, Rosbergen ICM, Jeffers MS, Corbett D. Is environmental enrichment ready for clinical application in human post-stroke rehabilitation? Front Behav Neurosci. 2018; 12 :135. doi: 10.3389/fnbeh.2018.00135 PMID: 33430635

3. BayneK. Environmental enrichment and mouse models: Current perspectives. Animal Model Exp Med. 2018; 1:82-90, doi: 10.1002/ame2.12015 PMID: 30891552

4. Cao L, Liu X, Lin EJ, Wang C, Choi EY, Riban V, Lin B, During MJ. Environmental and genetic activation of a brain-adipocyte BDNF/leptin axis causes cancer remission and inhibition. Cell. 2010;142:52-64. doi: 10.1016/j.cell.2010.05.029 PMID: 20603014

5. Fernandes MSS, Lacerda TR, Fidélis D, Santos GCJ, Filgueira TO, de Souza RF, Lagranha CJ, Lira FS, Castoldi A, Souto FO. Environmental enrichment in cancer as a

possible tool to combat tumor development: A Systematic Review. Int J Mol Sci.2023; 24:16516. doi: 10.3390/ijms242216326 PMID: 38003706

6. Grundy E. The living arrangements of elderly people.Rev Clin Gerontol. 1992; 2:353-361. doi: 10.1017/s0959259800003191 PMID: 12318668

7. Steptoe A, Shankar A, Demakakos P, Wardle J. Social isolation, loneliness, and all-cause mortality in older men and women. Proc Natl Acad Sci USA. 2013; 110:5797-5801.doi: 10.1073/pnas.1219686110 PMID: 23530191

8. Lobo RA, Gompel A. Management of menopause: a view towards prevention. Lancet Diabetes Endocrinol. 2022; 10:457-470, doi: 10.1016/S2213-8587(21)00269-2 PMID: 35526556

9. Yousefzadeh N, Kashfi K, Jeddi S, Ghasemi A. Ovariectomized rat model of osteoporosis: a practical guide. EXCLI J. 2020; 19:89-107. doi: 10.17179/excli2019-1990 PMID: 34121973

10. Minowa K, Rodriguez-Agudo D, Suzuki M, Muto Y, Hirai S, Wang Y, Su L, Zhou H, Chen Q, Lesnefsky EJ, Mitamura K, Ikegawa S, Takei H, Nittono H, Fuchs M, Pandak W.M, Kakiyama G. Insulin dysregulation drives mitochondrial cholesterol metabolite accumulation: initiating hepatic toxicity in nonalcoholic fatty liver disease. J Lipid Res. 2023; 64:100363. doi: 10.1016/j.jlr.2023.100363 PMID: 36966904

11. Funabashi D, Tsuchida R, Matsui T, Kita I, Nishijima T. Enlarged housing space and increased spatial complexity enhance hippocampal neurogenesis but do not increase physical activity in mice. Front Sports Act Living. 2023; 5:1203260. doi: 10.3389/fspor.2023.1203260 PMID: 37822972

12. Freid R, Hussein AI, Schlezinger JJ. Tributyltin protects against ovariectomy-induced trabecular bone loss in C57BL/6J mice with an attenuated effect in high fat fed mice. Toxicol Appl Pharmacol. 2021; 431:115736, doi: 10.1016/j.taap.2021.115736 PMID: 34619157

13. Faria SL, Faria OP, Menezes CS, de Gouvêa HR, de Almeida Cardeal M. Metabolic profile of clinically severe obese patients. Obes Surg. 2012; 22:1257-1262. doi: 10.1007/s11695-012-0651-y PMID: 22527595

14. Hatcher KM, Royston SE, Mahoney MM. Modulation of circadian rhythms through estrogen receptor signaling. Eur J Neurosci. 2020; 51:217-228. doi: 10.1111/ejn.14184 PMID: 30270552

15. Luo L, Liu M. Adipose tissue in control of metabolism. J Endocrinol. 2016; 231: R77-R99. doi: https://doi.org/10.1530/JOE-16-0211PMID: 27935822

16. Peppler WT, Townsend L.K, Knuth C.M, Foster M.T, Wright D.C. Subcutaneous inguinal white adipose tissue is responsive to, but dispensable for, the metabolic health benefits of exercise. Am J Physiol Endocrinol Metab. 2018; 314:E66-E77, doi: 10.1152/ajpendo.00226.2017 PMID: 28978546

17. Stachowiak G, Pertyński T, Pertyńska-Marczewska M. Metabolic disorders in menopause. Prz Menopauzalny. 2015; 14:59-64. doi: 10.5114/pm.2015.50000 PMID: 26327890

18. Hershon KS, Hirsch BR, Odugbesan O. Importance of postprandial glucose in relation to A1C and cardiovascular disease. Clin Diabetes. 2019; 37:250-259. doi: 10.2337/cd18-0040 PMID: 31371856

19. Hanssen NMJ, Kraakman MJ, Flynn MC, Nagareddy PR, Schalkwijk CG, Murphy AJ. Postprandial glucose spikes, an important contributor to cardiovascular disease in diabetes? Front Cardiovasc Med. 2020; 7 :570553. doi: 10.3389/fcvm.2020.570553 PMID: 33195459

20. Ogiso K, Shayo SC. Kawade S, Hashiguchi H, Deguchi T, Nishio Y. Repeated glucose spikes and insulin resistance synergistically deteriorate endothelial function and bardoxolone methyl ameliorates endothelial dysfunction. PLoS One. 2022; 17: e0263080, doi: 10.1371/journal.pone.0263080 PMID: 35073378

21. Medina-Contreras J, Villalobos-Molina R, Zarain-Herzberg A, Balderas-Villalobos J. Ovariectomized rodents as a menopausal metabolic syndrome model. A minireview, Mol Cell Biochem. 2020; 475:261-276, doi: 10.1007/s11010-020-03879-4 PMID: 32852713

22. Puolakkainen T, Rummukainen P, Pihala-Nieminen V, Ritvos O, Savontaus E, Kiviranta R. Treatment with soluble activin type IIB receptor ameliorates ovariectomy-

induced bone loss and fat gain in mice. Calcif Tissue Int. 2022; 110:504-517. doi: 10.1007/s00223-021-00934-0 PMID: 35024891

23. Beaupere C, Liboz A, Fève B, Blondeau B, Guillemain G. Molecular mechanisms of glucocorticoid-induced insulin resistance. Int J Mol Sci 2021; 22:623. doi: 10.3390/ijms22020623 PMID: 33435513

24. Ferris HA, Kahn CR. New mechanisms of glucocorticoid-induced insulin resistance: make no bones about it. J Clin Invest. 2012; 122:3854-3857. doi: 10.1172/JCI66180 PMID: 23093783

25. Wagenmaker ER, Moenter SM. Exposure to acute psychosocial stress disrupts the luteinizing hormone surge independent of estrous cycle alterations in female mice. Endocrinology. 2017; 158:2593-2602. doi: 10.1210/en.2017-00341 PMID: 28549157

26. Qiao S, Alasmi S, Wyatt A, Wartenberg P, Wang H, Candlish M, Das D, Aoki M, Grünewald R, Zhou Z, Tian Q, Yu Q, Götz V, Belkacemi A, Raza A, Ectors F, Kattler K, Gasparoni G, Walter J, Lipp P, Mollard P, Bernard DJ, Karatayli E, Karatayli SC, Lammert F, Boehm U. Intra-pituitary follicle-stimulating hormone signaling regulates hepatic lipid metabolism in mice. Nat Commun. 2023; 14:1098, doi: 10.1038/s41467-023-36681-z PMID: 36841874

27. Gong S, Miao YL, Jiao GZ, Sun MJ, Li H, Lin J, Luo MJ, Tan JH. Dynamics and correlation of serum cortisol and corticosterone under different physiological or stressful conditions in mice. PLoS One. 2015; 10:e0117503. doi: 10.1371/journal.pone.0117503 PMID: 25699675

28. Kinote A, Faria JA, Roman EA, Solon C, Razolli DS, Ignacio-Souza LM, Sollon CS, Nascimento LF, de Araújo TM, Barbosa AP, Lellis-Santos C, Velloso LA, Bordin S, Anhê GF. Fructose-induced hypothalamic AMPK activation stimulates hepatic PEPCK and gluconeogenesis due to increased corticosterone levels. Endocrinology. 2012; 153:3633-3645. doi: 10.1210/en.2012-1341 PMID: 22585831

29. Salgado MC, Metón I, Egea M, Baanante IV. Transcriptional regulation of glucose-6-phosphatase catalytic subunit promoter by insulin and glucose in the carnivorous fish, Sparus aurata. J Mol Endocrinol. 2004; 33:783-795, doi: 10.1677/jme.1.01552 PMID:

15591035

30. Wu L, Lu Y., Jiao Y, Liu B, Li S, Li Y, Xing F, Chen D, Liu X, Zhao J, Xiong X, Gu Y, Lu J, Chen X, Li X. Paternal psychological stress reprograms hepatic gluconeogenesis in offspring. Cell Metab. 2016; 23:735-743. doi: 10.1016/j.cmet.2016.01.014 PMID: 26908462

31. DeFronzo RA, Felig P, Ferrannini E, Wahren J. Effect of graded doses of insulin on splanchnic and peripheral potassium metabolism in man. Am J Physiol. 1980; 238: E421-427. doi: 10.1152/ajpendo.1980.238.5.E421 PMID: 6990783

32. Svenson KL, Paigen B. Recommended housing densities for research mice: filling the gap in data-driven alternatives. FASEB J. 2019; 33:3097-3111. doi: 10.1096/fj.201801972R PMID: 30521372

33. Yanai S, Semba Y, Endo S. Remarkable changes in behavior and physiology of laboratory mice after the massive 2011 Tohoku earthquake in Japan. PLoS One. 2012; 7:e44475, doi: 10.1371/journal.pone.0044475 PMID: 22957073

34. Konopka AR, Harber MP. Skeletal muscle hypertrophy after aerobic exercise training. Exerc Sport Sci Rev. 2014; 42 :53-61. doi: 10.1249/JES.0000000000000007 PMID: 24508740

35. Irwin ML, Yasui Y, Ulrich CM, Bowen D, Rudolph RE, Schwartz RS, Yukawa M, Aiello E, Potter JD, McTiernan A. Effect of exercise on total and intra-abdominal body fat in postmenopausal women: a randomized controlled trial. JAMA. 2003; 289:323-330.doi: 10.1001/jama.289.3.323 PMID: 12525233

36. Friedman M. Hyperthermia as a manifestation of stress. Res Publ Assoc Res Nerv Ment Dis. 1949; 29:433-444. PMID: 14854307

37. Kokolus KM, Capitano ML, Lee CT, Eng JW, Waight JD, Hylander BL, Sexton S, Hong CC, Gordon CJ, Abrams SI, Repasky EA. Baseline tumor growth and immune control in laboratory mice are significantly influenced by subthermoneutral housing temperature. Proc Natl Acad Sci USA. 2013; 110:20176-20181. doi: 10.1073/pnas.1304291110 PMID: 24248371

Figure Legends

Figure 1. Housing environments and body weight changes

(A) Experimental schedule. (B) Images of three different housing environments, (Left) EE, (Middle) SC, and (Right) IS environments. (C,D) Body weight at week 1 (C) and week 4 (D). (E) Body weight changes in (C) and (D). In all groups, n = 10. Experiments were repeated at least three times with similar results. All values are shown as the means ± SE. One-way ANOVA and Tukey's multiple comparisons test were used; ***P* < 0.01

SE, standard error; ANOVA, analysis of variance; OVX, ovariectomized; SC, standard conditions; EE, enriched environment; IS, isolated conditions

Figure 2. Measurements of feed intake, physical activity, and body temperature

(A) Daily feed intake per mouse. (B) Momentum per mouse. (C) Area under the curve for (B). (D) Body temperature during week 3. (E) Area under the curve for (D). (F,G) Body temperature at 01:00 (F) and 13:00 (G). Red: EE, Black: SC, Blue: IS. Experiments were repeated at least three times with similar results. All values are shown as the means ± SE. One-way ANOVA and Tukey's multiple comparisons test were used; **P* < 0.05, ***P* < 0.01, *** *P* < 0.001, **** *P* < 0.001

SE, standard error; ANOVA, analysis of variance; OVX, ovariectomized; SC, standard conditions; EE, enriched environment; IS, isolated conditions

Figure 3. Measurements of white adipose tissue weight and adipocyte size

(A) Gonadal white adipose tissue (gWAT) weight at week 5 (n = 14-15). (B) Representative images of H&E staining in gWAT. Scale bars: 100 μm. (C) Quantification of the adipocyte area (area per adipocyte, μm^2) performed using a microscope at 20× magnification in four random fields per section (n = 5). The experiments were repeated at least three times, and similar results were obtained. All values are shown as the means ± SE. One-way ANOVA and Tukey's multiple comparison tests were used; **P* < 0.05, ***P* < 0.01, *** *P* < 0.001, **** *P* < 0.001

SE, standard error; ANOVA, analysis of variance; OVX, ovariectomized; SC, standard

conditions; EE, enriched environment; IS, isolated conditions

Figure 4. Measurement of metabolic parameters and serological analysis

(A) GTT in sham (left) and OVX (right) mice (n = 5). (B) Area under the curve for (A). (C) Blood glucose level 15 minutes after glucose injection during GTT. (D) Fasting blood glucose level. (E) Leisure blood glucose level. (F) Leisure serum insulin level. (G) Fasting serum insulin level. (H) HOMA-IR value. (I) Serum corticosterone level. (J) PTT in sham (left) and OVX (right) mice (n = 5). (K) Area under the curve for (J). (L–V) Serological tests for total protein (L), Albumin (M), LDL-cholesterol (N), HDL-cholesterol (O), T-cholesterol (P), triglyceride (Q), sodium (R), chloride (S), calcium (T), inorganic phosphorus (U), and potassium (V). Experiments were repeated at least three times with similar results. All values are shown as the means ± SE. One-way ANOVA and Tukey's multiple comparisons test were used; *$P < 0.05$, **$P < 0.01$, *** $P < 0.001$

SE, standard error; ANOVA, analysis of variance; OVX, ovariectomized; SC, standard conditions; EE, enriched environment; IS, isolated conditions; HOMA-IR, homeostasis model assessment of insulin resistance; glucose (GTT) and pyruvate (PTT) tolerance tests; L/HDL, low/high density lipoprotein

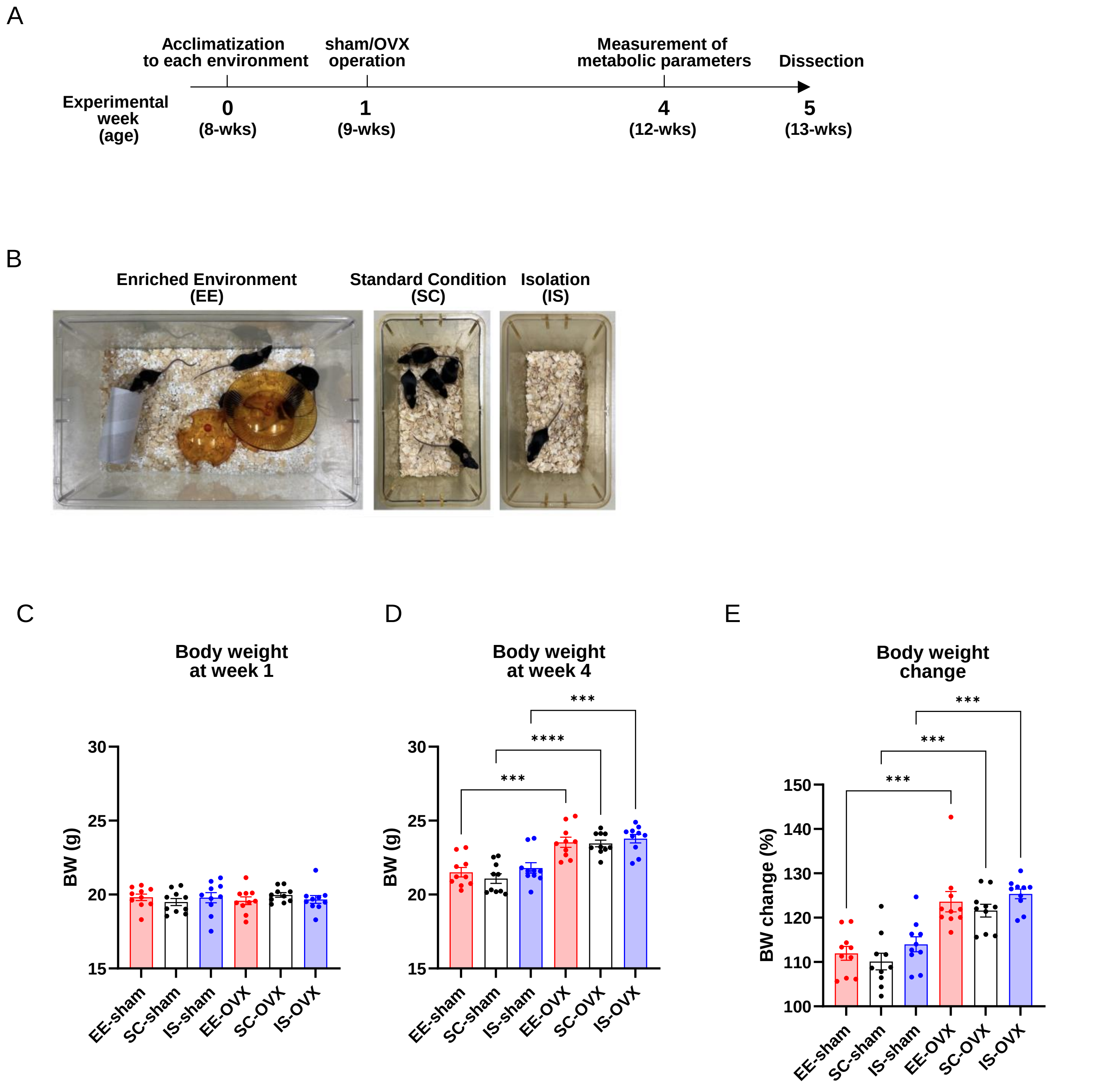
A
Acclimatization to each environment
sham/OVX operation
Measurement of metabolic parameters
Dissection
Experimental week (age)
0 (8-wks)
1 (9-wks)
4 (12-wks)
5 (13-wks)
B
Enriched Environment (EE)
Standard Condition (SC)
Isolation (IS)
C
Body weight at week 1
BW (g)
30
25
20
15
EE-sham
SC-sham
IS-sham
EE-OVX
SC-OVX
IS-OVX
D
Body weight at week 4
***
****
***
BW (g)
30
25
20
15
EE-sham
SC-sham
IS-sham
EE-OVX
SC-OVX
IS-OVX
E
Body weight change
***
***
***
BW change (%)
150
140
130
120
110
100
EE-sham
SC-sham
IS-sham
EE-OVX
SC-OVX
IS-OVX

Fig. 1 Ju *et al*.

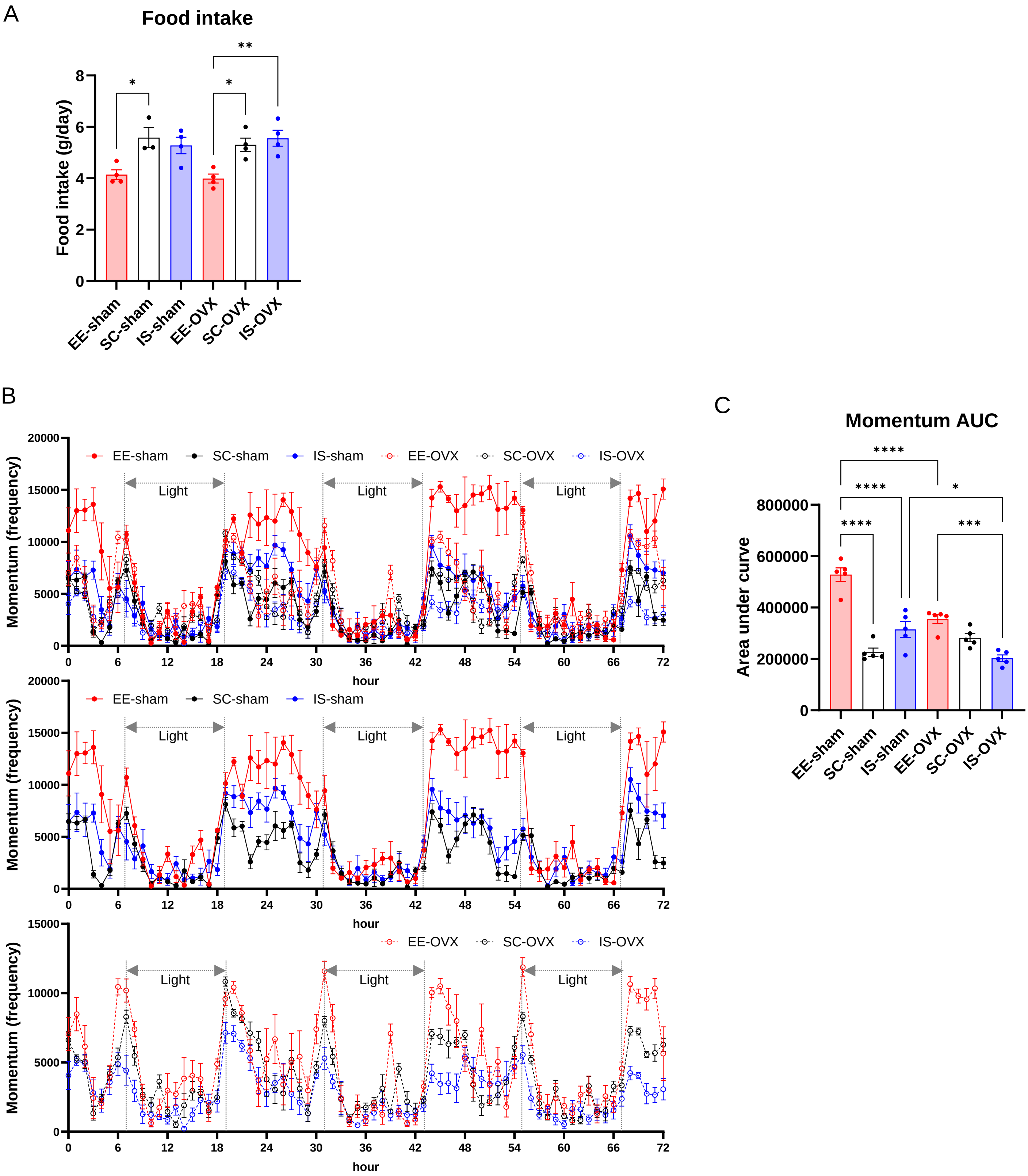

Fig. 2 Ju *et al*. (Continued)

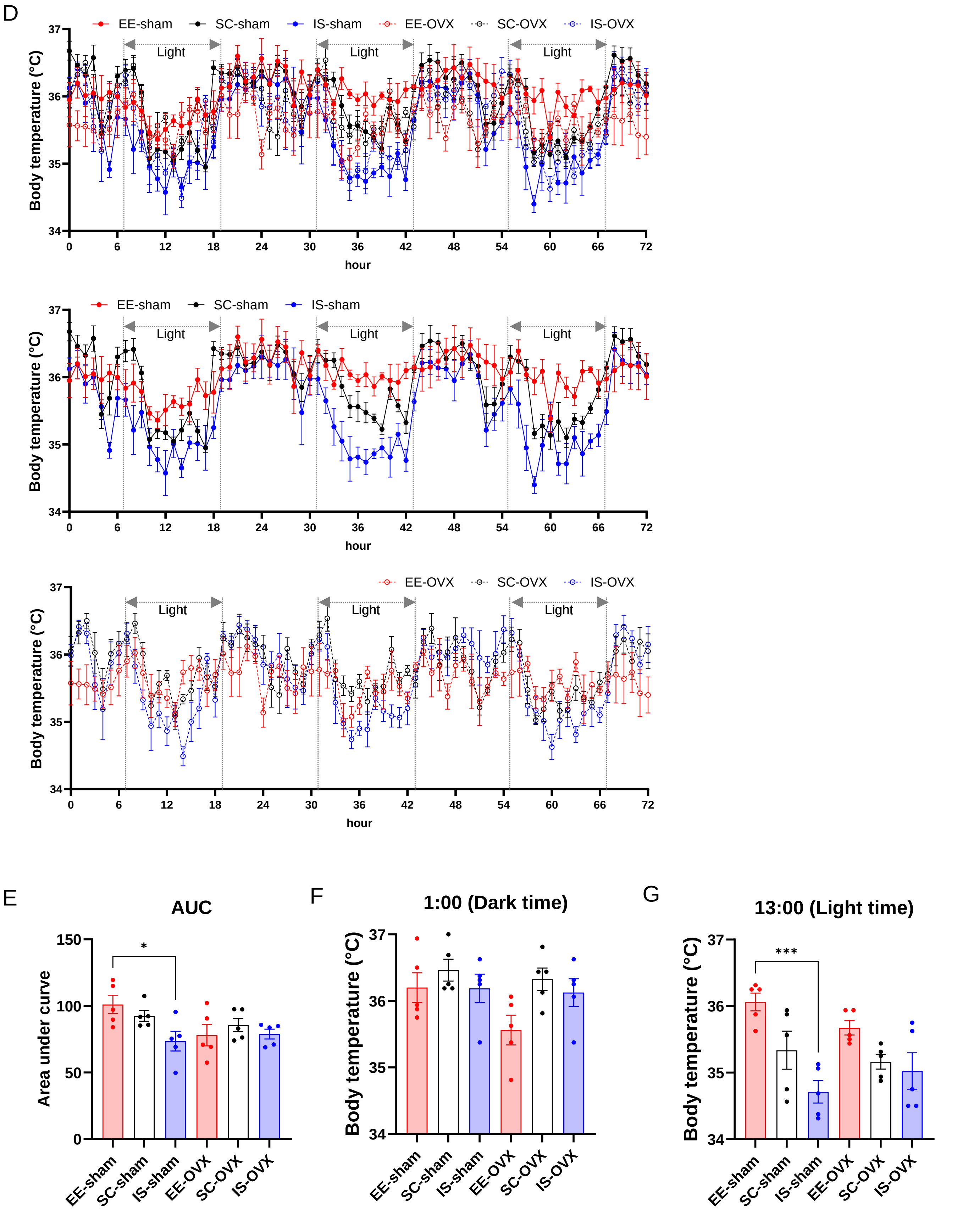
D
EE-sham
SC-sham
IS-sham
EE-OVX
SC-OVX
IS-OVX
Light
Body temperature (°C)
hour
E
AUC
Area under curve
*
F
1:00 (Dark time)
Body temperature (°C)
G
13:00 (Light time)
Body temperature (°C)
***

Fig. 2 Ju *et al.*

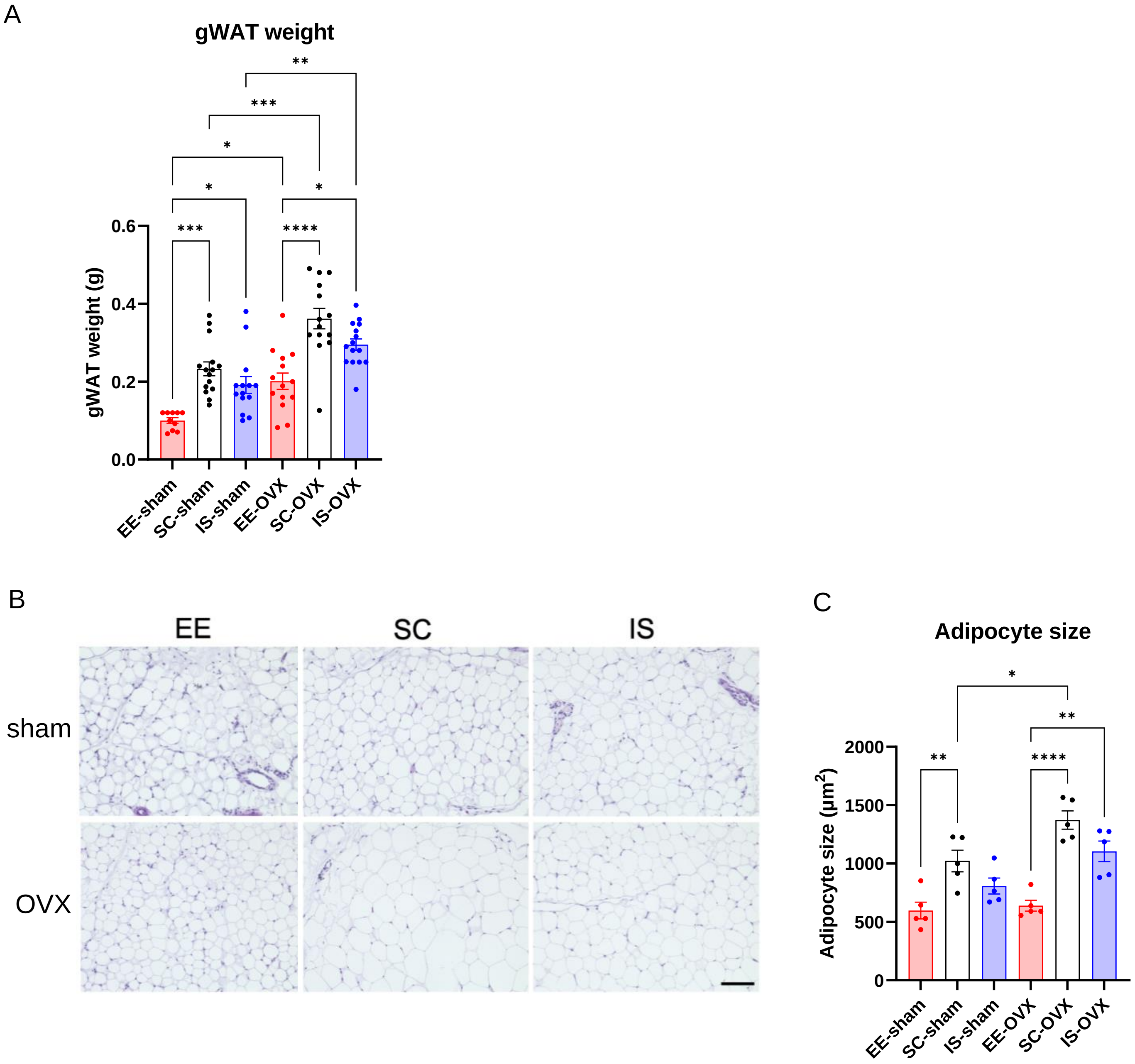
A
gWAT weight
gWAT weight (g)
0.0
0.2
0.4
0.6
***
*
*
***
**
****
*
EE-sham
SC-sham
IS-sham
EE-OVX
SC-OVX
IS-OVX
B
EE
SC
IS
sham
OVX
C
Adipocyte size
Adipocyte size (μm²)
0
500
1000
1500
2000
**
****
**
*
EE-sham
SC-sham
IS-sham
EE-OVX
SC-OVX
IS-OVX

Fig. 3 Ju *et al*.

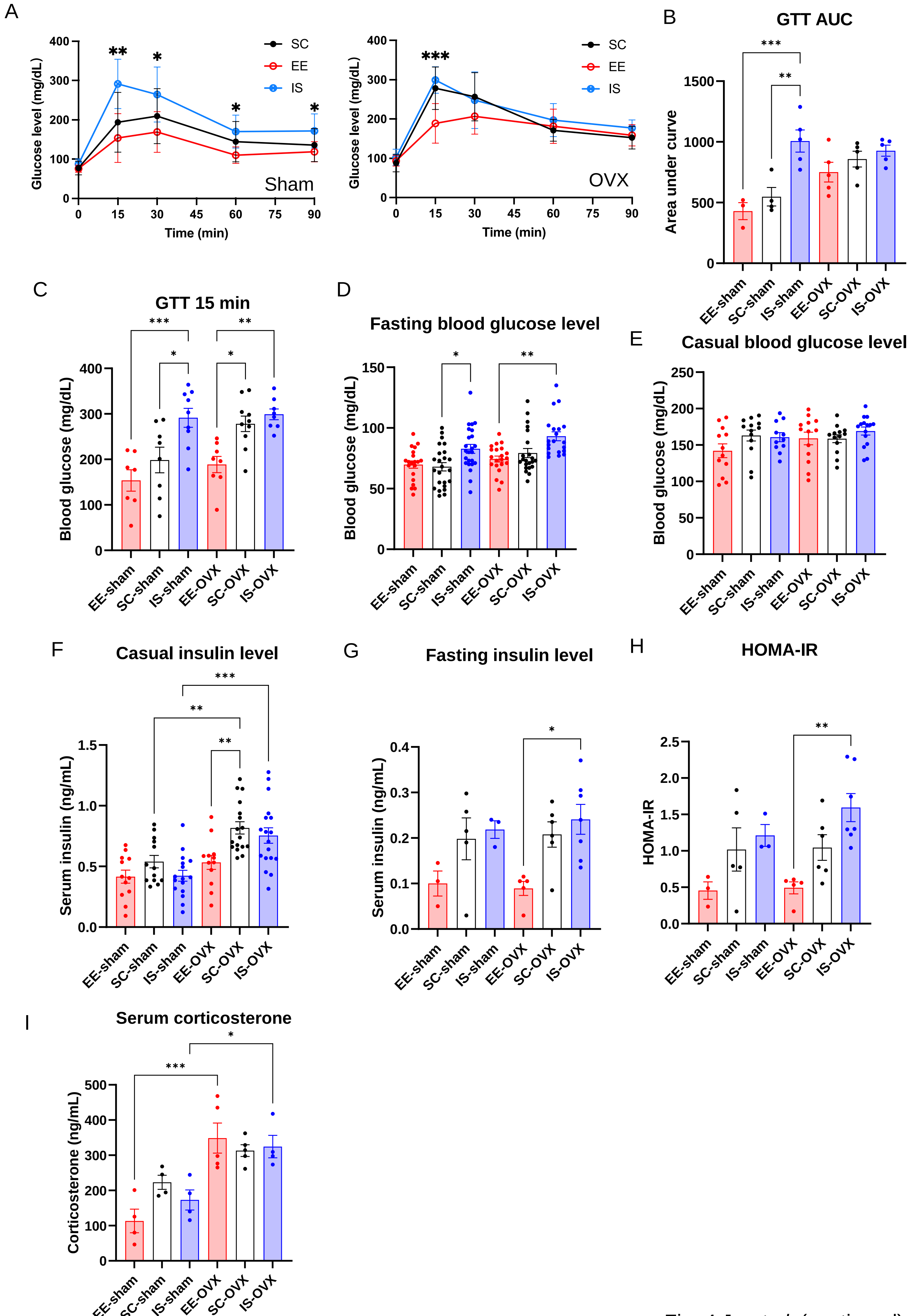

Fig. 4 Ju *et al*. (continued)

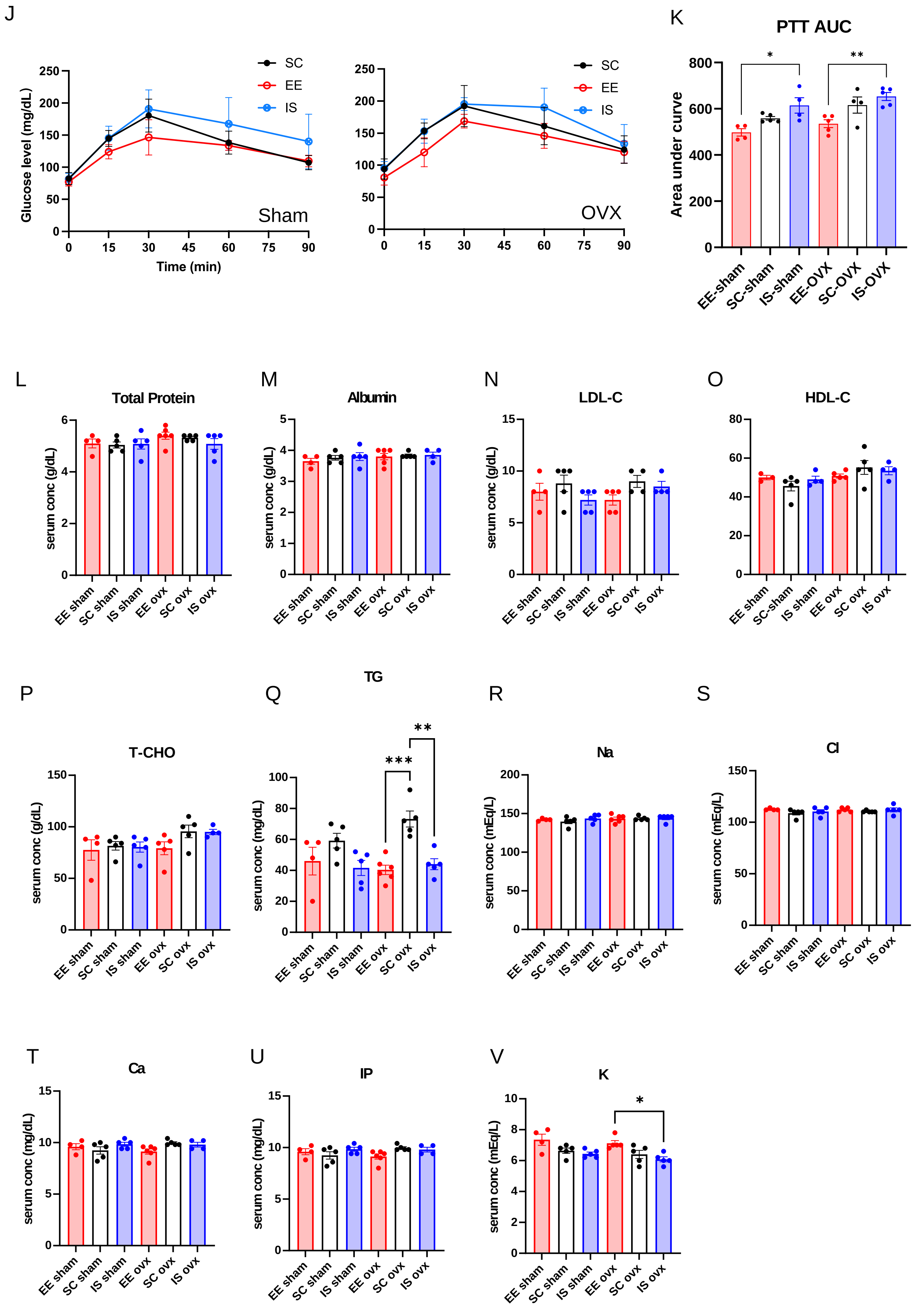

J
K
PTT AUC
Glucose level (mg/dL)
Time (min)
SC
EE
IS
Sham
OVX
Area under curve
EE-sham
SC-sham
IS-sham
EE-OVX
SC-OVX
IS-OVX
L
Total Protein
M
Albumin
N
LDL-C
O
HDL-C
P
T-CHO
Q
TG
R
Na
S
Cl
T
Ca
U
IP
V
K
serum conc (g/dL)
serum conc (mg/dL)
serum conc (mEq/L)
EE sham
SC sham
IS sham
EE ovx
SC ovx
IS ovx

Fig. 4 Ju *et al*.
