# Supplementary material for: The impact of environmental enrichment on energy metabolism in ovariectomized mice
Source: PLoS One. 2025 Apr 2;20(4):e0320180. doi: 10.1371/journal.pone.0320180 (PMC11964458; doi:10.1371/journal.pone.0320180)
Supplement: Attachment — Submitted filename: Response to Reviewers_PLoS One.docx [file pone.0320180.s004.docx]

Response to Reviewers:

We sincerely appreciate the thorough analyses and constructive suggestions provided by the reviewer, which have been very helpful in guiding us to improve our study further. We hope the editor and the reviewer will concur with us after reading the enclosed point-to-point response that we have addressed all the raised concerns satisfactorily and that the revised manuscript is now suitable for its publication as a Research Article in PLOS ONE.

To Reviewer #1:

*Dear Authors,
Strictly follow my comments/correction in the attached file. In general:
1. at the end of the introduction section, need of the study should be ideally raised followed by the hypothesis of the study.*

We appreciate the reviewer’s comment. We added a statement of the need for this research and a hypothesis at the end of the “Introduction” section (page2, Line 65-68) in the revised manuscript.

*2. in statistical analysis, add mathematical model for better understanding of data analysis.*

In agreement with the reviewer’s suggestion, we revised the “Materials and Methods” section (page 4, Line 126-132) in the revised manuscript.

*3. in results, add actual p-value of each result*

According to the reviewer’s suggestion, we added respective actual p-values to all graphs in which significant differences were obtained. We added p-values in the “Figure Legends” section (lines 156, 190, and 218) in the revised manuscript.

*4. in conclusion, add limitations of the study*

According to the reviewer’s suggestion, we added limitations of the study in the “Conclusion” section (page 14, Line 325-329) in the revised manuscript.

We also revised the manuscript about the following minor points that the reviewer kindly pointed out.

*5. Page 4, Line 91-93, in the original manuscript, which experimental design was followed?*

We revised the sentence to “The mice at 8-week-old were randomized by body weight and assigned to either the enriched environment (EE), the standard condition (SC), or the isolated condition (IS)” in the “Materials and Methods” section (page 3, Line 79-80) in the revised manuscript.

*6. Page 15, Line 376-379, in the original manuscript, Add reference.*

The appropriate reference #10 was added to the “Material & methods” section on page 3, line 98, and to the "References" section on p15, lines 276-379, in the revised manuscript.
